# Genetic characterization of influenza A (A/H3N2) viruses reveals antigenic drift in receptor binding domain and possible vaccine mismatch in strains circulating in Riyadh, Saudi Arabia, 2024–2025

**DOI:** 10.1186/s12879-026-12928-0

**Published:** 2026-02-19

**Authors:** Shatha Ata Abdulgader, Ibrahim M. Aziz, Abdulhadi M. Abdulwahed, Mohamed A. Farrag, Reem M. Aljowaie, Abdulaziz M. Almuqrin, Noorah A. Alkubaisi, Fahad N. Almajhdi

**Affiliations:** 1 Department of Clinical Laboratory Sciences, College of Applied Medical Sciences, King Saud University, Riyadh, 12372 Saudi Arabia; 2 Department of Botany and Microbiology, College of Science, King Saud University, Riyadh, 11451 Saudi Arabia

**Keywords:** Vaccine compatibility, IAV, Phylogenetic analysis, Glycosylation sites, Molecular surveillance

## Abstract

**Introduction:**

Influenza A/H3N2 viruses undergo continuous antigenic evolution, necessitating ongoing surveillance for informed vaccine strain selection. This study characterized the molecular epidemiology of A/H3N2 viruses circulating in Riyadh, Saudi Arabia, during the winter season of 2024-2025 and assessed their compatibility with current vaccine strains.

**Methods:**

Nasopharyngeal samples (NPAs) (n=363) were collected from patients presenting with influenza-like illness at King Khalid University Hospital in Riyadh. Influenza A/H3N2 detection and subtyping were performed using RT-PCR. Complete hemagglutinin (HA) and neuraminidase (NA) genes sequencing was conducted on confirmed A/H3N2 strains (n=7), followed by phylogenetic analysis, amino acid substitution mapping, and glycosylation site prediction.

**Results:**

Of 363 samples tested, 110 (30.3%) were positive for influenza A, with 42 (38.2%) identified as A/H3N2 and 68 (61.8%) as A/H1N1pdm09. Phylogenetic analysis revealed that all seven sequenced A/H3N2 strains belonged to clade 2a.3a.1, which is identical to the current vaccine strain clade. However, molecular analysis identified four amino acid substitutions in the HA glycoprotein that distinguished circulating strains from the A/H3N2 vaccine strain A/Croatia/10136RV/2023. The NA gene was homologous to the study isolates, indicating a possible match with the vaccine. Notably, all study strains showed the same N-glycosylation sites that are present in the vaccine strain.

**Conclusions:**

While phylogenetic clade compatibility suggests potential vaccine effectiveness, the observed amino acid differences highlight the importance of continued molecular surveillance to monitor antigenic drift and assess vaccine performance in the Saudi Arabian population.

**Supplementary Information:**

The online version contains supplementary material available at 10.1186/s12879-026-12928-0.

## Introduction

Influenza viruses are highly contagious pathogens and the leading cause of acute febrile respiratory illness worldwide. They impose a major public-health and economic burden, triggering seasonal epidemics and, at times, devastating pandemics [1–3]. Several subtypes of influenza A virus (IAV) have caused global outbreaks in the past century, including the 1918 H1N1, 1957 H2N2, 1968 A/H3N2, and 2009 A/H1N1 pandemics [4]. The ability of IAV to cross species barriers and to evolve continuously through genetic change has enabled the emergence of novel antigenic variants such as H5N1, H7N9, H9N2, H5N8, and H7N7 [5, 6].

Two main mechanisms drive the evolutionary of IAV: antigenic drift and antigenic shift. Antigenic drift arises from point mutations that accumulate gradually within the viral genome, particularly at the antigenic sites of the surface glycoproteins hemagglutinin (HA) and neuraminidase (NA). Because the viral RNA-dependent RNA polymerase lacks proofreading activity, these mutations occur frequently and can alter viral tropism, virulence, replication efficiency, and antigenicity [7]. In contrast, antigenic shift results from reassortment of the segmented viral RNA during co-infection, generating new genome arrangements with unique antigenic properties and the potential to cause pandemics [8, 9].

Among IAV subtypes, A/H3N2 viruses have demonstrated remarkable antigenic drift since their first detection in 1968 [10]. The World Health Organization (WHO) has consequently updated the A/H3N2 vaccine component more than 28 times [11]. Despite these efforts, vaccine effectiveness often remains suboptimal; for example, efficacy during the 2016–2017 season was only 28–42% across age groups [12]. Such limited protection underscores the need for continuous genetic and antigenic monitoring to guide timely vaccine reformulation [13].

Saudi Arabia represents a unique setting for respiratory-virus transmission owing to its annual mass gatherings (Hajj and Umrah) and the movement of millions of foreign workers [14]. These dynamics can accelerate viral importation, circulation, and mutation. Although several local studies have described influenza A/H3N2 activity in previous years [15, 16], information on its molecular epidemiology and genetic diversity remains limited—particularly for the 2024–2025 season. Our previous surveillance in Riyadh (2014–2020) showed that 48.8% of IAV isolates were A/H3N2 [17]. Also, our recently and a more recent surveillance in Riyadh (2020–2023) revealed that A/H3N2 subtype was found in 9.21% of IAV isolates, which overcame the number of A/H1N1 isolates 7.89% [18]. However, comprehensive molecular characterization of circulating strains during the recent 2024–2025 season remains absent, creating a critical knowledge gap for evidence-based vaccine policy.

This study specifically aimed to (1) determine the prevalence of A/H3N2 viruses in Riyadh during 2024–2025; (2) characterize the complete *HA* and *NA* genes sequences of circulating strains; (3) assess phylogenetic relationships and clade classification; (4) identify amino-acid substitutions within key antigenic sites; (5) evaluate N-glycosylation patterns; and (6) determine vaccine-strain compatibility for the strains identified from Saudi population. Understanding these molecular patterns will provide critical insight for national vaccination policy, future vaccine-strain selection, and preparedness for potential influenza epidemics in Saudi Arabia.

## Materials and methods

### Ethical approval

The study was conducted in accordance with the Declaration of Helsinki and approved by the Research Ethics Committee at King Saud University, Riyadh, Saudi Arabia (Institutional Review Board Nos. E-24-9609 and E-25-9609, approved in November 2023 and May 2025, respectively). All NPAs that tested positive for IAV were handled in accordance with ethical standards, ensuring patient confidentiality and appropriate medical follow-up as per King Saud University regulations.

### Acquisition of clinical samples

A total of 363 NPAs were collected from individuals presenting with influenza-like illness (ILI) at King Khalid University Hospital, Riyadh, between September 2024 and February 2025. Patients exhibiting symptoms such as fever, cough, sore throat, runny nose, muscle or body aches, headache, or fatigue were enrolled after providing informed consent. Individuals who had been vaccinated with seasonal influenza vaccines were excluded from the study. Each specimen was mixed with 2 mL of virus minimum essential medium (MEM) transport medium (Gibco, Invitrogen, Grand Island, NY, USA) and transported under refrigerated conditions to the Virology Research Laboratory, College of Science, King Saud University. Samples were briefly vortexed for 15 s, centrifuged at 1,000× g for 10 min at 4 °C, aliquoted, and stored at − 80 °C until further analysis.

### Typing, detection and sequencing of IAV

#### Detection and typing

Viral RNA was extracted using the QIAamp Viral RNA Mini Kit (Qiagen, Hilden, Germany) following the manufacturer’s instructions. Detection and subtyping of IAV were carried out using the One-Step Ahead RT-PCR Kit with Taq High-Fidelity DNA Polymerase (Qiagen, Hilden, Germany; Cat. No. 220213). The reaction was performed on a GeneAmp 9700 Thermal Cycler (Applied Biosystems, USA) under the following cycling conditions: reverse transcription at 50 °C for 30 min, initial denaturation at 95 °C for 15 min, followed by 35 cycles of denaturation at 94 °C for 15 s, annealing at 52 °C for 30 s, and extension at 72 °C for 2 min. A final extension was performed at 72 °C for 10 min. PCR products were visualized by electrophoresis on a 1% agarose gel stained with ethidium bromide and compared against a 100 bp DNA ladder (Qiagen, Germany).

#### Amplification of full-length *HA* and *NA* genes

The *HA* and *NA* genes of A/H3N2 strains were amplified using the same kit and thermal protocol described above, with two sets of overlapping primers to obtain the complete gene sequences. Primer sequences and PCR conditions for *HA* and *NA* gene amplification are detailed in Table 1. All primers were designed based on conserved regions and validated using reference strains before clinical application. Seven representative A/H3N2 isolates (*n* = 7; three from 2024 and four from 2025) were selected for full-length *HA* and *NA* genes sequencing based on availability of sufficient remaining sample volume, successful initial subtyping, and even temporal distribution across the 2024–2025 season. This selection approach ensured that the sequenced isolates were of adequate quality for complete gene amplification and sequencing while representing viruses circulating throughout the entire study period. Sequencing was performed by Macrogen Inc. (Seoul, South Korea). Raw sequence data were edited using BioEdit v7.0 (Ibis Biosciences, Carlsbad, CA, USA) and assembled using the EditSeq and MegAlign modules of Lasergene software v3.18 (DNAStar, Madison, WI, USA). All generated sequences were submitted to GenBank under accession numbers EPI_ISL_653577, EPI_ISL_653578, EPI_ISL_653579, EPI_ISL_653580, EPI_ISL_653581, EPI_ISL_653582, EPI_ISL_653583 for *HA* gene. EPI_ISL_653552, EPI_ISL_653553, EPI_ISL_653554, EPI_ISL_653555, EPI_ISL_653556, EPI_ISL_653557, EPI_ISL_653558.

**Table 1:** Primers used in this study for detection, typing, and sequencing of influenza A/H3N2

	Type/subtype	Gene	Primer name	Sequence 5’-3’	Product Size (bp)	Ref.
Employed primers for identification	IAV		M30F2/08	ATGAGYCTTYTAACCGAGGTCGAAACG	244	[16]
			M264R3/08	TGGACAAANCGTCTACGCTGCAG		
Primers employed for typing	(A/H1N1) Pdm09		HKU-SWF	TGAGCTCAGTGTCATCATTTGA	174	[16]
			HKU-SWR	TGCTGAGCTTTGGGTATGAA		
	A/H3N2		H3A1F6	AAGCAGGGGATAATTCTATTAACC	1127	
			H3A1R1	GTCTATCATTCCCTCCCAACCATT		
Used primers for sequencing	A/H3N2	*HA*	H3A1F6	AAGCAGGGGATAATTCTATTAACC	55 °C	
			H3A1R1	GTCTATCATTCCCTCCCAACCATT		
			HA828F	ACGAAGTGGGAAAAGCTCAATA	50 °C	[19]
			HA1778R	AGTAGAAACAAGGGTGTTTT		
	A/H3N2	*NA*	NA-1 F	GAGCAAAAGCAGGAGTAAAG	50 °C	
			NA787R	TGACAATGTGCTAGTATGAAC		
			NA636F	AGATAGTGTTGTTTCATGGTC	50 °C	
			NA1413R	AGTAGAAACAAGGAGTTTTT		

**Table 2:** Distributions of clinical specimens categorized by season, gender, and age

		No. of samples *N* (%)	Positive for IBV *N* (%)	Positive for IAV *N* (%)	Positive for	
					A/H1N1pdm09 *N* (%)	A/H3N2 *N* (%)
Total		363	68 (18.7)	110 (30.3)	68 (61.8)	42 (38.2)
Season	2024	166 (45.7)	18 (26.0)	47 (28.3)	38 (80.8)	9 (19.2)
	2025	197 (54.3)	50(73.5)	63 (31.9)	30 (47.6)	33 (52.4)
Gender	Male	176 (48.4)	20 (28.9)	45 (25.5)	29 (64.4)	16 (35.5)
	Female	187 (51.5)	48 (70.5)	65 (34.7) ^a^	39 (60) ^a^	26 (39.4)
Age in years	0–4	121 (33.3)	12 (17.6)	27 (22.3)	13 (48.1)	14 (51.8)
	5–14	88 (24.2)	24(35.2)	29 (32.9)	20 (68.9)	9 (31.0)
	15–64	101 (27.8)	26 (38.2)	33 (32.6) ^b^	16 (48.4)	17 (51.5) ^b^
	≥ 65	53 (14.6)	6 (8.8)	21 (39.6)	19 (90.4)	2 (9.5)

Note: the data provided are expressed in numerical values (%), an indicates a significantly greater value (*P* < 0.05) compared to males. b Indicates a significantly greater value (*P* < 0.05) in comparison to the age groups of 0–4, 5–14, and those aged 65 years and older

### Sequence and phylogenetic analysis

Raw sequence data were edited and assembled using BioEdit v7.0 (Ibis Biosciences, Carlsbad, CA, USA) and Lasergene v3.18 (DNAStar, Madison, WI, USA). Multiple sequence alignment of the complete *HA* and *NA* genes was performed using the ClustalW algorithm implemented in the MegAlign program. The analysis compared the A/H3N2 strains from Riyadh with 100 local and international reference and vaccine strains retrieved from GISAID and GenBank databases (see Supplementary Table S1). The 100 reference sequences were selected as the top BLAST hits from GISAID and GenBank databases based on high sequence coverage (> 95%), high sequence identity, recency (primarily strains collected in 2023–2025), and representation of diverse geographic regions. Only high-quality, complete sequences passing strict quality control were included, specifically those without ambiguous bases in key regions and with proper *HA*/*NA* gene annotation. Study sequences were compared to the vaccine strain A/Croatia/10136RV/2023 and the reference strain A/New York/392/2004 (used as consensus for variation mapping). Sequence variations, including amino acid substitutions, insertions/deletions (indels), and nucleotide divergences, were evaluated. In addition, potential N-linked and O-linked glycosylation sites were predicted using NetNGlyc 1.0 [20] and NetOGlyc 3.1 [21], respectively. All amino acid positions and substitutions are reported according to the standard H3 numbering scheme (mature HA1 numbering, as used in contemporary H3N2 literature and WHO vaccine strain annotations).Phylogenetic trees for both the *HA* and *NA* genes were constructed using the neighbor-joining method implemented in MEGA v11 software (Pennsylvania State University, University Park, PA, USA), with 1,000 bootstrap replicates to assess branch reliability.

### Statistical analysis

Statistical analysis was performed using IBM SPSS v26.0. Categorical variables (positivity rates by sex, age group, and collection year) were compared using Fisher’s exact test. Post-hoc pairwise comparisons were conducted with the Z-test and Bonferroni correction for multiple testing. The primary hypotheses tested were whether infection rates differed significantly across demographic groups and between the 2024 and 2025 periods (*p* < 0.05 considered significant).

## Results

### Detection and typing of influenza A/H3N2

Among the 363 NPAs analyzed, 110 (30.3%) were positive for IAV, and 68 (18.7%) were confirmed cases of influenza B (IBV). Although these IBV-positive samples were part of the original screening dataset, the current study’s genomic characterization was limited to H3N2-positive samples.

Of these 110 positive IAV samples, 68 (61.8%) were identified as A/H1N1pdm09 and 42 (38.2%) as A/H3N2. Samples were categorized into two collection periods: 2024 and 2025. In 2024, A/H1N1pdm09 was predominant (80.8%) whereas A/H3N2 accounted for only 19.2%. However, A/H3N2 prevalence increased markedly in 2025, representing 52.4% of positive IAV cases.

Of the total specimens, 176 (48.4%) were from males and 187 (51.5%) from females. A higher infection rate was recorded among females (34.7%) compared with males (25.5%, *p* < 0.05). Age distribution analysis revealed that individuals aged 15–64 years had the highest positivity rate (32.6%), which was significantly higher than the rates in the 0–4, 5–14, and ≥ 65-year age groups (*p* < 0.05). Detailed demographic and seasonal distributions are presented in Table 2.

### Nucleotide and amino-acid variation in *HA* and *NA* Genes

Complete *HA* (1,701 nt) and *NA* (1,410 nt) gene sequences were obtained from seven representative A/H3N2 isolates (three from 2024 and four from 2025) were compared with 100 globally circulating A/H3N2 strains retrieved from the GISAID and GenBank databases using the consensus sequence (A/New York/392/2004). (Table S1). The sequence analysis indicated that the nucleotide identity varied from 97.75 to 98.56% (*HA*), whereas the *NA* identity was observed to be between 97.23 and 98.65%.

**Fig. 1:**
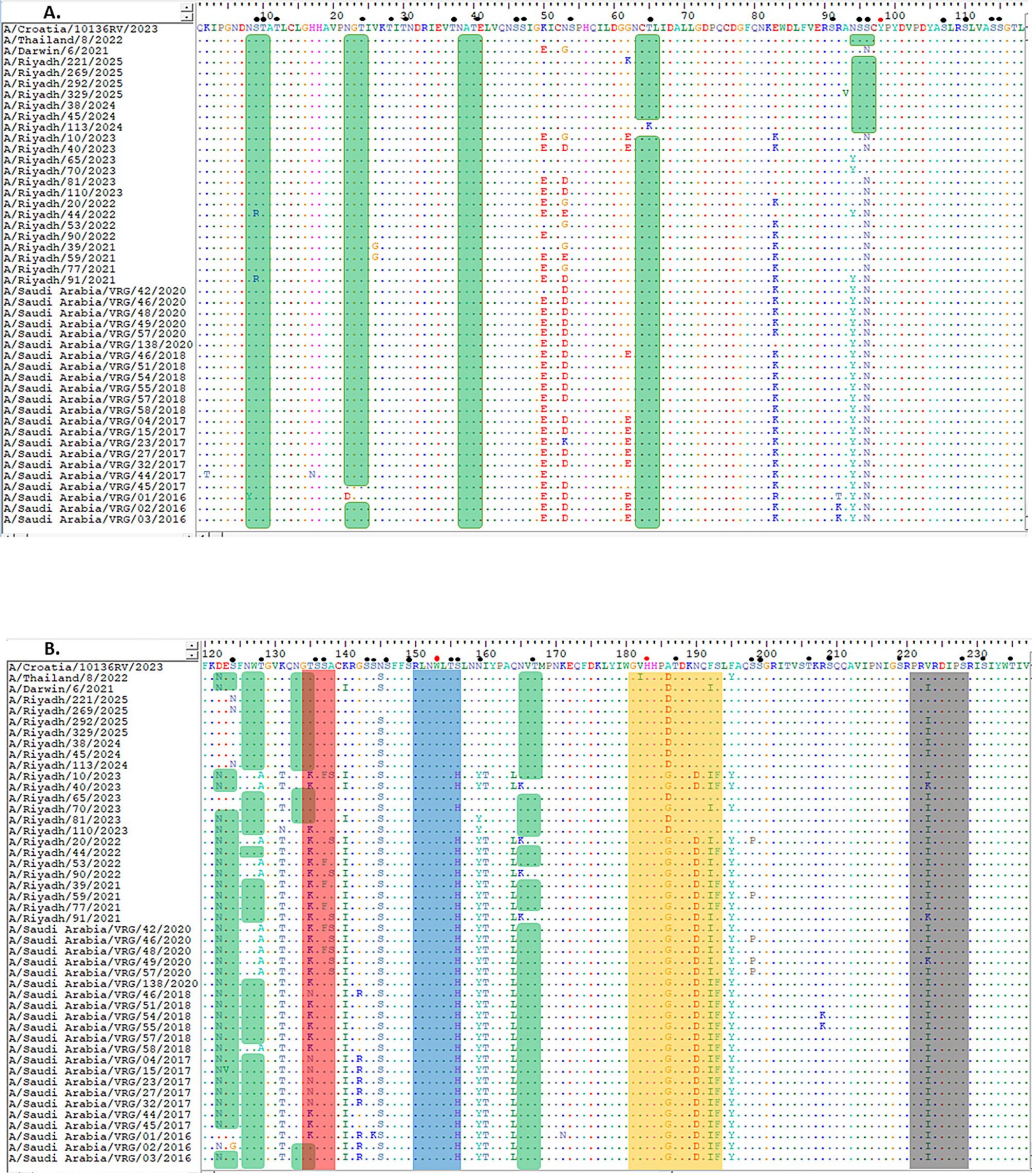

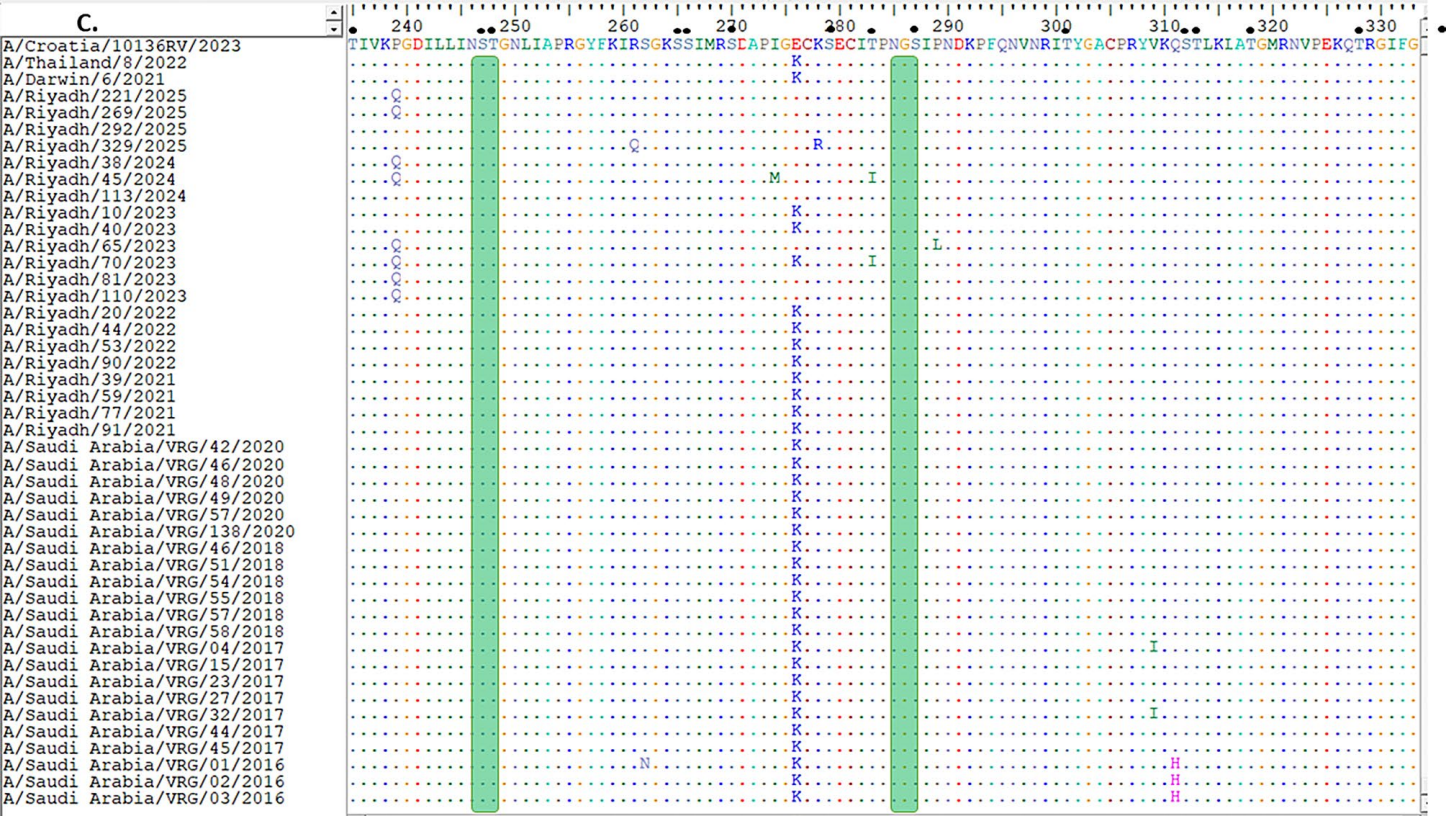
HA1 domain of *HA* gene: Alignment and Comparison of A/Riyadh A/H3N2 Strains with the Vaccine Strain (A/Croatia/10136RV/2023, A/Thailand/8/2022, and A/Darwin/6/2021), and other local circulating strains. (**A**) amino acid residues from 1 to 118, (**B**) from 119 to 237, and (**C**) from 235 to 334. Identical amino acids are represented by colored dots, whereas variations in amino acids are denoted using capital letters. The enclosed red rectangle represents the 130-loop (residues 134–138); the enclosed blue rectangle represents the 150-loop (residues 150–156); the enclosed orange rectangle represents the 190-helix (residues 181–193); and the enclosed gray rectangle represents the 220-loop (residues 221–228). Red dots indicate conserved residues. Predicted N-linked glycosylation sites are enclosed in green rectangles. Small, filled black circles correspond to predicted O-linked glycosylation sites

**Fig. 2:**
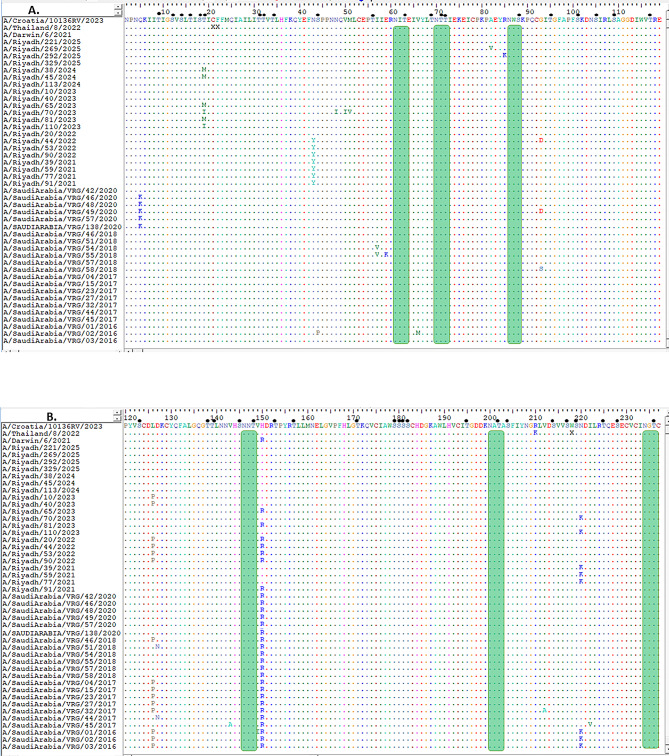

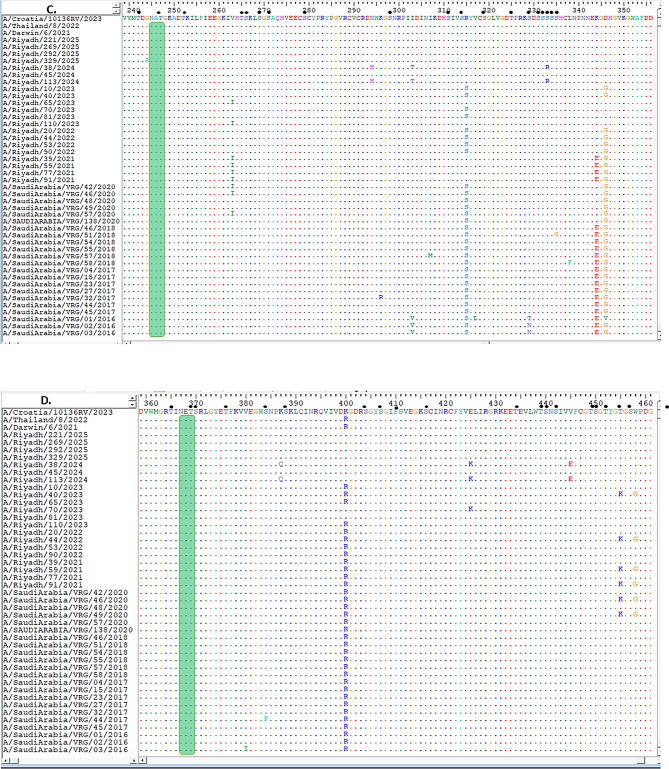
Describe the *NA* gene sequence of study strains A/H3N2 in alignment and comparison with vaccine strain and other local circulating strains, (**A**) amino acid residues from 1 to 119, (**B**) from 120 to 237, (**C**) from 239 to 356, and (**D**) from 358 to 461. Identical amino acids are shown as colored dots, while variations in the amino acids are represented by uppercase letters. The locations of N-glycosylation sites are marked with green rectangles, and potential O-glycosylation sites are signified by black dots

Comparison of the HA1 domain between the current vaccine strain A/Croatia/10136RV/2023 and the A/Riyadh isolates revealed four amino-acid differences (N145S, A186D, V223I, P239Q) (Fig. 1A, B and C). On the other hand, the *NA* gene of the current vaccine strain A/Croatia/10136RV/2023 was homologous to the study isolates (Fig. 2A, B, C and D).

**Fig. 3:**
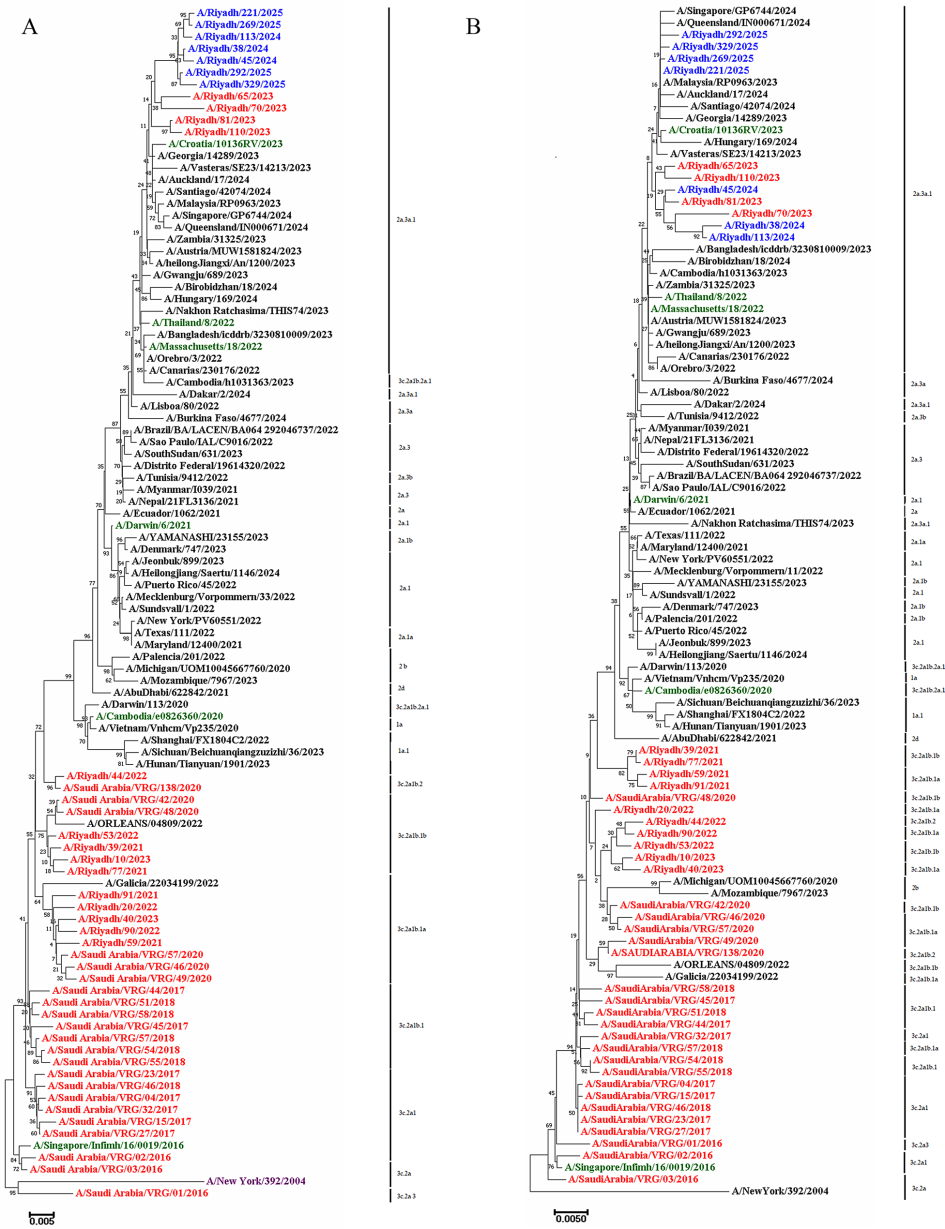
A phylogenetic tree for A/H3N2 constructed from nucleotide sequences of the (**A**) *HA* gene and (**B**) *NA* gene. The study strains of A/H3N2 are highlighted in blue, green color showed vaccine strains, local circulating strains in previous years are denoted in red, and reference strains are shown in purple

The aim of such comparison is to compare current circulating strains with vaccine to ensure the effectiveness of current vaccine strain against highly mutated gene of A/H3N2 of circulating strains. Also, since the vaccine are designed for highly conserved region of the virus, it is important to monitor these differences to reduce risk of transmutation, lowering the burden of influenza disease, and it can also help in predicting early pandemic of the virus in the country.

Several identical sequences connected to antigenicity within the receptor-binding domain (RBD), including the 130-loop, 150-loop, 190-helix, and 220-loop, were observed when the HA1 of the Riyadh isolates was compared to the vaccine strains (A/Croatia/10136RV/2023) (Fig. 1B). Most of the isolates in the study demonstrated a consistent change in the A186D and V223I amino acid changes within the 190-helix and 220-loop, respectively, in contrast to the vaccine strain (Fig. 1B).

The mutations mentioned above were observed not only in our A/H3N2 Riyadh strains but also in sequences obtained from various countries globally; however, they were absent in the A/H3N2 vaccine strain A/Croatia/10136RV/2023. In fact, these mutations in our strains were situated within or adjacent to recognized antigenic sites, which may influence antigenic drifts and the recognition of the virus by the vaccine.

#### HA cleavage site analysis

The HA cleavage site motif in all seven Riyadh A/H3N2 strains was ³²⁰PEKQTR↓GL³²⁷ (H3 numbering), featuring a single arginine residue. This monobasic cleavage site is characteristic of low-pathogenic seasonal human influenza A viruses and identical to that of the vaccine strain A/Croatia/10136RV/2023, indicating no evidence of enhanced pathogenicity potential.

### Glycosylation-site analysis

Analysis of the HA1 open reading frame predicted 10–11 N-glycosylation sites (positions 8, 22, 38, 63, 94, 126, 133, 165, 246, and 285). O-glycosylation analysis revealed potential modification sites at positions 55–59 within the HA1 domain, similar to the vaccine strain (Fig. 1A, B and C).

The NA protein possessed nine N-glycosylation sites (positions 61, 70, 86, 146, 200, 233, 245, and 367) consistent with the vaccine strain. O-glycosylation sites ranged from 70 to 72, also comparable to those of the vaccine strain (Fig. 2A, B, C and D).

### Phylogenetic analysis

Phylogenetic trees constructed for both the *HA* and *NA* genes demonstrated that all Riyadh A/H3N2 strains clustered within clade 2a.3a.1, the same clade as the WHO-recommended vaccine strain A/Croatia/10136RV/2023. This finding indicates high genetic similarity between the circulating and vaccine strains (Fig. 3Aand 3B).

Sequence homology with the vaccine strain was approximately 16%, supporting the phylogenetic inference of relatedness. The Riyadh isolates were distinct from earlier local strains (2014–2020) and (2020–2023) but closely related to recent international strains such as A/Georgia/14,289/2023, A/Vasteras/SE23/14,213/2023, and A/Auckland/17/2024. These patterns suggest recent introduction of genetically distinct viruses into the region, which is facilitated by international air travelling activity.

## Discussion

Influenza A/H3N2 viruses undergo continuous antigenic evolution through point mutations (antigenic drift), which necessitates ongoing molecular surveillance to guide vaccine strain selection and assess potential mismatches [7, 11, 13]. The present study provides updated molecular data on the circulation, genetic variation, clade distribution, and vaccine compatibility of influenza A/H3N2 viruses in Riyadh during the 2024–2025 winter season. Of 363 nasopharyngeal samples collected from patients presenting with influenza-like illness at King Khalid University Hospital, 110 (30.3%) were positive for IAV, with 42 (38.2%) identified as A/H3N2 and 68 (61.8%) as A/H1N1pdm09. A clear temporal shift was observed: A/H1N1pdm09 predominated in 2024, while A/H3N2 prevalence rose sharply in 2025, accounting for 52.4% of IAV-positive cases in that period. This alternating subtype dominance pattern is consistent with previous reports from the Middle East and North Africa, where A/H1N1pdm09 and A/H3N2 have repeatedly alternated in predominance [15, 18–20]. The rapid shift observed here likely reflects the high mutation rate and strong antigenic drift characteristic of A/H3N2 viruses [21]. Demographically, adults aged 15–64 years showed the highest positivity rate, while females exhibited a significantly higher infection rate than males (*p* < 0.05). These patterns are broadly in line with prior Saudi studies [22, 23], although differences in age and sex distribution compared to our 2020–2023 data [18], suggest that local transmission dynamics may vary between seasons.

HA glycoprotein mediates receptor binding and viral entry [24]. Key RBD elements include the 130-loop (134–138), 150-loop (150–156), 190-helix (181–193), 220-loop (221–228), and conserved residues Tyr98, Trp153, His183 [25–28]. Antigenic drift often affects these regions or major antigenic sites (Ca, Cb, Sa, Sb), enabling immune escape [29]. Comparison of seven Riyadh strains with the vaccine strain A/Croatia/10136RV/2023 revealed four HA1 substitutions: N145S, A186D, V223I, and P239Q. A186D and V223I map to the 190-helix and 220-loop, potentially altering receptor binding and antigenicity [24, 25, 30]. All strains carried the same N- glycosylation both in HA and NA glycoproteins, while having four mutations site in *HA* gene compared to vaccine strain. Despite clade-level similarity to the vaccine, these HA changes suggest possible antigenic differences. Residues 186 and 223 are recurrent mutation hotspots globally and locally, likely driven by immune pressure [31–33]. These substitutions, near antigenic sites, may influence antigenic drift and vaccine recognition, facilitating reinfection [21, 24, 30]. Continuous local molecular surveillance is crucial to monitor such changes and guide vaccine updates.

To assess evolutionary trends of A/H3N2 in Riyadh, current strains were compared with the historical reference strain A/New York/392/2004 and with strains from previous local studies conducted during 2014–2020 and 2020–2023. A total of 108 and 60 amino-acid mutation sites were identified in the *HA* and *NA* genes, respectively, including 12 HA and five NA mutations unique to the 2024–2025 strains. Despite this accumulated genetic variation, phylogenetic analysis placed all current isolates within clade 2a.3a.1, the same clade as the vaccine strain A/Croatia/10136RV/2023, representing a shift from our earlier findings in which Riyadh strains clustered within 3 C.2a lineages [17], or subclades 3 C.2a1b.1a and 3 C.2a1b.1b [18], often diverging from vaccine-related clades. Phylogenetic clustering further suggests that international travel plays a significant role in shaping the genetic diversity of circulating A/H3N2 viruses in Saudi Arabia, as several isolates showed closer relatedness to recent international strains (e.g., A/Georgia/14289/2023, A/Västerås/SE23/14213/2023, A/Auckland/17/2024) than to earlier local Riyadh lineages [14, 34–36]. Given Saudi Arabia’s extensive inbound travel for employment, tourism, and mass religious gatherings such as Hajj and Umrah, repeated introductions of genetically distinct viruses are likely, potentially altering local viral ecosystems, increasing selective pressure, and influencing population immunity [34–36]. Although clade-level concordance with the vaccine strain is reassuring, phylogenetic similarity does not guarantee antigenic equivalence, as minor substitutions in HA or NA can significantly affect antibody recognition [12]. While predicted N- and O-linked glycosylation sites were largely conserved between circulating strains and the vaccine strain, the four genetic mutations in *HA* gene may contribute to antigenic differences [37–41]. Overall, these findings suggest that the 2024–2025 A/H3N2 vaccine component provides adequate genetic coverage for strains circulating in Riyadh, while underscoring the necessity of continuous molecular and serological surveillance to monitor ongoing viral evolution and inform timely vaccine updates.

The data support that the 2024–2025 A/H3N2 vaccine component likely provided adequate genetic coverage for strains circulating in Riyadh. However, the identified amino-acid substitutions compared to vaccine strain highlight the virus’s ongoing evolution and the need for year-round molecular surveillance. This study is limited by its focus on only the *HA* and *NA* gene segments, whereas full-genome sequencing would offer deeper insights into overall genetic diversity, potential reassortment events, and the role of internal genes in viral fitness. Additional constraints include the relatively small number of sequenced isolates (*n* = 7) and single-center sampling from one tertiary-care hospital in Riyadh. It is important to note that these samples were collected from a single tertiary-care hospital in Riyadh; therefore, the observed prevalence rates may not be fully representative of the broader city or national population and should be interpreted with caution. This limits generalizability to the wider Saudi population. Furthermore, the absence of antigenic characterization such as HI testing restricts definitive conclusions about vaccine match and effectiveness. Future multicenter studies incorporating larger sample sizes, complete-genome sequencing, and serological/phenotypic assays will be essential to more comprehensively monitor viral evolution and support evidence-based influenza vaccine policy in the region.

## Conclusions

This study provides updated molecular insights into the circulation and genetic diversity of influenza A/H3N2 viruses in Riyadh, Saudi Arabia, during the 2024–2025 influenza season. Among the 363 samples analyzed, A/H3N2 accounted for 42 (38.2%) of all IAV detections and increased in frequency during 2025. Phylogenetic analysis demonstrated that all local isolates belonged to clade 2a.3a.1, the same clade as the current WHO-recommended vaccine strain A/Croatia/10136RV/2023, indicating a high level of vaccine–strain compatibility. Nevertheless, four amino-acid substitutions in the HA glycoprotein and sequence homology of NA glycoprotein underline the virus’s continuing molecular evolution.

Although the overall vaccine match appears favorable, the observed mutations of our strains compared to vaccine strain warrant continued genomic surveillance and functional evaluation to assess their effects on antigenicity and vaccine performance. Expanding molecular surveillance programs across multiple Saudi regions and integrating serological assays such as HI tests will be essential for early detection of emerging variants and for supporting evidence-based updates of seasonal vaccine formulations.

## Supplementary Information

Below is the link to the electronic supplementary material.

Supplementary Material 1

## Data Availability

The datasets generated and analyzed during the current study are available from the corresponding author upon reasonable request. The sequence data reported in this study have been deposited in GenBank under accession numbers PV653577–PV653583 for the HA gene and PV653652–PV653658 for the NA gene.

## References

[1] Uyeki TM, Hui DS, Zambon M, Wentworth DE, Monto. AS Influenza Lancet. 2022;400:693–706. 36030813 10.1016/S0140-6736(22)00982-5 PMC9411419

[2] Morens DM, Folkers GK. Fauci AS.The challenge of emerging and re-emerging infectious diseases.Nature. 2004; 430: 242–9. 10.1038/nature02759 PMC7094993 15241422

[3] Dunning J, Thwaites RS, Openshaw PJ. Seasonal and pandemic influenza: 100 years of progress, still much to learn.Mucosal immunology. 2020; 13: 566–73. 10.1038/s41385-020-0287-5 PMC7223327 32317736

[4] Palese P. Influenza: old and new threats.Nature medicine. 2004; 10: S82–7. 10.1038/nm1141 15577936

[5] Tate M. Highly pathogenic avian H5N8 influenza viruses: should we be concerned? In: Taylor & Francis; 2018. pp. 20–1. 10.1080/21505594.2017.1386832 PMC5801643 28968185

[6] Westerhuis B, Ten Hulscher H, Jacobi R, van Beek J, Koopmans M, Rimmelzwaan G, Meijer A, van Binnendijk. R.Specific memory B cell response in humans upon infection with highly pathogenic H7N7 avian influenza virus.Scientific reports. 2020; 10: 3152. 10.1038/s41598-020-60048-9 PMC7035254 32081953

[7] Hay AJ, Gregory V, Douglas AR, Lin YP. The evolution of human influenza viruses. Philos Trans R Soc B. 2001;356:1861. 10.1098/rstb.2001.0999 PMC1088562 11779385

[8] Schäffr JR, Kawaoka Y, Bean WJ, Süss J, Senne D, Webster RG. Origin of the pandemic 1957 H2 influenza A virus and the persistence of its possible progenitors in the avian reservoir.Virology. 1993; 194: 781–8. 10.1006/viro.1993.1319 7684877

[9] Webby R, Webster RG. Emergence of influenza A viruses.Philosophical Transactions of the Royal Society of London. Ser B. 2001;356:1817. 10.1098/rstb.2001.0997 PMC1088557 11779380

[10] Alymova IV, York IA, Air GM, Cipollo JF, Gulati S, Baranovich T, Kumar A, Zeng H, Gansebom S. McCullers JA.Glycosylation changes in the globular head of H3N2 influenza hemagglutinin modulate receptor binding without affecting virus virulence. Sci Rep. 2016;6:36216. 27796371 10.1038/srep36216 PMC5086918

[11] Allen JD. Ross TM.H3N2 influenza viruses in humans: viral mechanisms, evolution, and evaluation. Human Vaccines & Immunotherapeutics. 2018; 14: 1840–1847. 10.1080/21645515.2018.1462639 PMC6149781 29641358

[12] Melidou A, Gioula G, Exindari M, Ioannou E, Gkolfinopoulou K, Georgakopoulou T, Tsiodras S. Papa A.Ιnfluenza A(H3N2) genetic variants in vaccinated patients in northern Greece. J Clin Virol. 2017;94:29–32. 28734139 10.1016/j.jcv.2017.07.003

[13] Blackburne BP, Hay AJ, Goldstein RA. Changing selective pressure during antigenic changes in human influenza H3. PLoS Pathog. 2008;4:e1000058. 10.1371/journal.ppat.1000058 PMC2323114 18451985

[14] Al Shammari BR. Sequence and phylogenetic analysis of influenza virus (H1N1pdm2009) circulating in Riyadh. Saudi Arabia J Pure Appl Microbiol. 2024; 18.

[15] Awadalla ME, Alkadi H, Alarjani M, Al-Anazi AE, Ibrahim MA, ALOhali TA, Enani M, Alturaiki W. Alosaimi B.Moderately low effectiveness of the influenza quadrivalent vaccine: Potential mismatch between circulating strains and vaccine strains.Vaccines. 2023; 11: 1050. 10.3390/vaccines11061050 PMC10304586 37376439

[16] Who. CC.WHO information for the molecular detection of influenza viruses.World Health Organisation. 2017.

[17] Dudin GA, Aziz IM, Alzayed RM, Ahmed A, Hussain T, Somily AM, Alsaadi MM. Almajhdi FN.Genetic diversity and evolutionary kinetics of influenza A virus H3N2 subtypes circulating in Riyadh. Saudi Arabia Vaccines. 2023;11:702. 36992286 10.3390/vaccines11030702 PMC10054866

[18] Alkubaisi NA, Aziz IM, Farrag MA, Aljowaie RM, Alsaleh AN, Alanazi FN, Almajhdi FN. Evolution and Vaccine Strain Match of HA and NA Genes of Influenza A/H3N2 Subtype in Riyadh, Saudi Arabia, 2020–2023. Vaccines. 2025;13:1184. 10.3390/vaccines13121184 PMC12737434 41441651

[19] Al-Dorzi HM, Alsafwani ZA, Alsalahi E, Aljulayfi AS, Alshaer R, Alanazi S, Aldossari MA, Alsahoo DA. Khan R.Patients with influenza admitted to a tertiary-care hospital in Riyadh between 2018 and 2022: characteristics, outcomes and factors associated with ICU admission and mortality.BMC Pulmonary Medicine. 2024; 24: 464. 10.1186/s12890-024-03281-6 PMC11414087 39300448

[20] Al Khatib HA, Al Thani AA, Gallouzi I, Yassine HM.Epidemiological and genetic characterization of pH1N1 and H3N2 influenza viruses circulated in MENA region during 2009–2017.BMC Infectious Diseases. 2019; 19: 314. 10.1186/s12879-019-3930-6 PMC6458790 30971204

[21] Nelson N MI, Edelman L, Spiro D, Boyne AR, Bera J, Halpin R, Sengamalay N, Ghedin E, Miller MA, Simonsen L. Correction: Molecular Epidemiology of A/H3N2 and A/H1N1 Influenza Virus during a Single Epidemic Season in the United States. PLoS Pathog. 2008;4:annotatione1013711391941–d13919931391943. 10.1371/journal.ppat.1000133 PMC2495036 18725925

[22] Althaqafi A, Farahat F, Alsaedi A, Alshamrani M, Alsaeed MS, AlhajHussein B, El-Kafrawy SA, Azhar EI. Molecular detection of influenza A and B viruses in four consecutive influenza seasons 2015–16 to 2018–19 in a tertiary center in Western Saudi Arabia. J Epidemiol global health. 2021;11:208–15. 10.2991/jegh.k.210427.001 PMC8242120 33969948

[23] Gomaa MR, Badra R, El Rifay AS, Kandeil A, Kamel MN, Abo Shama NM, El-Shesheny R, Barakat AB, Ali MA, Kayali G. Incidence and seroprevalence of seasonal influenza a viruses in Egypt: results of a community-based cohort study. Influenza and Other Respiratory Viruses. 2022; 16: 749–55. 10.1111/irv.12974 PMC9178055 35179306

[24] Xing L, Chen Y, Chen B, Bu L, Liu Y, Zeng Z, Guan W, Chen Q, Lin Y, Qin K. Antigenic drift of the hemagglutinin from an influenza A (H1N1) pdm09 clinical isolate increases its pathogenicity in vitro. Virol Sin. 2021;36:1220–7. 34106413 10.1007/s12250-021-00401-y PMC8188537

[25] Yang H, Chen L-M, Carney P, Donis RO, Stevens J. Structures of receptor complexes of a North American H7N2 influenza hemagglutinin with a loop deletion in the receptor binding site. PLoS Pathog. 2010;6:e1001081. 20824086 10.1371/journal.ppat.1001081 PMC2932715

[26] Skehel JJ, Wiley DC. Receptor binding and membrane fusion in virus entry: the influenza hemagglutinin. Annu Rev Biochem. 2000;69:531–69. 10966468 10.1146/annurev.biochem.69.1.531

[27] Mair CM, Ludwig K, Herrmann A, Sieben C. Receptor binding and pH stability—How influenza A virus hemagglutinin affects host-specific virus infection. Biochimica et Biophysica Acta (BBA)-Biomembranes. 2014; 1838: 1153–68. 10.1016/j.bbamem.2013.10.004 24161712

[28] Tzarum N, De Vries RP, Zhu X, Yu W, McBride R, Paulson JC, Wilson IA. Structure and receptor binding of the hemagglutinin from a human H6N1 influenza virus.Cell host & microbe. 2015; 17: 369–76. 10.1016/j.chom.2015.02.005 PMC4374348 25766295

[29] Samal SK. Structural vaccinology approaches to enhance efficacy, stability, and delivery of protective antigens. In: Reverse vaccinology: Elsevier; 2024. pp. 217–235.

[30] Raymond DD, Bajic G, Ferdman J, Suphaphiphat P, Settembre EC, Moody MA, Schmidt AG, Harrison SC. Conserved epitope on influenza-virus hemagglutinin head defined by a vaccine-induced antibody.Proceedings of the National Academy of Sciences. 2018; 115: 168–173. 10.1073/pnas.1715471115 PMC5776812 29255041

[31] Peng W, Liu H, Wang X, Li C, Huang S, Qi S, Hu Z, Xu X, Jiang H, Duan J. Analysis of Epidemiological and Evolutionary Characteristics of Seasonal Influenza Viruses in Shenzhen City from 2018 to 2024.Viruses. 2025; 17: 798. 10.3390/v17060798 PMC12197669 40573388

[32] Song Y, Zhang X, Ji J, Li L, Zhou Y, Li P, Ren G, Lv S, Zhang X. Yan Y.Genetic characteristics of influenza A/H3N2 virus in Jiaxing, China (2019–2024). Virol J. 2025;22:1–14. 41188942 10.1186/s12985-025-02974-6 PMC12584209

[33] Koel BF, Burke DF, Bestebroer TM, Van Der Vliet S, Zondag GC, Vervaet G, Skepner E, Lewis NS, Spronken MI. Russell CA.Substitutions near the receptor binding site determine major antigenic change during influenza virus evolution.Science. 2013; 342: 976–979. 10.1126/science.1244730 24264991

[34] Agustiningsih A, Indalao IL, Pangesti KA, Sukowati CHC, Ramadhany RM. Characterization of Influenza A/H3N2 Virus Isolated from Indonesian Hajj and Umrah Pilgrims 2013 to 2014.Life. 2023; 13: 1100. 10.3390/life13051100 PMC10221221 37240745

[35] Alfelali M, Khandaker G, Booy R, Rashid H. Mismatching between circulating strains and vaccine strains of influenza: Effect on Hajj pilgrims from both hemispheres. Hum Vaccines Immunotherapeutics. 2016;12:709–15. 10.1080/21645515.2015.1085144 PMC4964745 26317639

[36] Sevilla NL. Germs on a Plane: The Transmission and Risks of Airplane-Borne Diseases. Transp Res Rec. 2018;2672:93–102. 34285452 10.1177/0361198118799709 PMC8282645

[37] Chang D, Hackett WE, Zhong L, Wan X-F, Zaia J. Measuring site-specific glycosylation similarity between influenza a virus variants with statistical certainty.Molecular &. Cell Proteom. 2020;19:1533–45. 10.1074/mcp.RA120.002031 PMC8143645 32601173

[38] Tate MD, Job ER, Deng Y-M, Gunalan V, Maurer-Stroh S, Reading PC. Playing hide and seek: how glycosylation of the influenza virus hemagglutinin can modulate the immune response to infection.Viruses. 2014; 6: 1294–316. 10.3390/v6031294 PMC3970151 24638204

[39] Gallagher P, Henneberry J, Sambrook J, Gething M. Glycosylation requirements for intracellular transport and function of the hemagglutinin of influenza virus. J Virol. 1992;66:7136–45. 1331514 10.1128/jvi.66.12.7136-7145.1992 PMC240399

[40] Kim JI, Park. M-S.N-linked glycosylation in the hemagglutinin of influenza A viruses.Yonsei medical journal. 2012; 53: 886. 10.3349/ymj.2012.53.5.886 PMC3423856 22869469

[41] Alcala E. Advancements in Influenza Glycobiology: Impacts in Influenza A Virus Evolution. Fitness, and Vaccine Development: University of Missouri-Columbia; 2024.

