# Supplementary material for: Genetic characterization of influenza A (A/H3N2) viruses reveals antigenic drift in receptor binding domain and possible vaccine mismatch in strains circulating in Riyadh, Saudi Arabia, 2024–2025
Source: BMC Infect Dis. 2026 Feb 19;26:636. doi: 10.1186/s12879-026-12928-0 (PMC13020053; doi:10.1186/s12879-026-12928-0)
Supplement: Supplementary file 1 — Supplementary Material 1 [file 12879_2026_12928_MOESM1_ESM.docx]

**Table S2**: List of H3N2 strains included in sequence and phylogenetic analysis

| **No.** | **Strain** | **Origin** | **Gisaid Accession No.** | | **Gisaid Clade** |
| --- | --- | --- | --- | --- | --- |
|  |  |  | **HA** | **NA** |  |
|  | A/New York/392/2004 | USA/Ref. | EPI252225 | EPI79013 | 3c.2a |
|  | A/Singapore/Infimh/16/0019/2016 | Singapore | EPI1381186 | EPI1381185 | 3c.2a1 |
|  | A/Cambodia/e0826360/2020 | CambodiA | EPI1843589 | EPI1843588 | 3c.2a1b.2a.1 |
|  | A/Darwin/113/2020 | Australia | EPI1733843 | EPI1733842 | 3c.2a1b.2a.1 |
|  | A/Darwin/6/2021 | Australia | EPI1885402 | EPI1885401 | 2a.1 |
|  | A/Michigan/UOM10045667760/2020 | USA | EPI2095215 | EPI2095213 | 2b |
|  | A/ORLEANS/04809/2022 | France | EPI1999181 | EPI1999180 | 3c.2a1b.1b |
|  | A/Galicia/22034199/2022 | Spain | EPI2287489 | EPI2287488 | 3c.2a1b.1a |
|  | A/YAMANASHI/23155/2023 | Japan | EPI3045768 | EPI3045767 | 2a.1b |
|  | A/New_York/PV60551/2022 | USA | EPI2433730 | EPI2433728 |  |
|  | A/Jeonbuk/899/2023 | South Korea | EPI2716104 | EPI2716103 | 2a.1 |
|  | A/Santiago/42074/2024 | Chile | EPI3502603 | EPI3502602 | 2a.3a.1 |
|  | A/Dakar/2/2024 | Senegal | EPI3426277 | EPI3426271 | 2a.3a.1 |
|  | A/Zambia/31325/2023 | Zambia | EPI2900250 | EPI2900246 | 2a.3a.1 |
|  | A/Bangladesh/icddrb/3230810009/2023 | Bangladesh | EPI3501178 | EPI3501176 | 2a.3a.1 |
|  | A/Singapore/GP6744/2024 | Singapore | EPI3468466 | EPI3468465 | 2a.3a.1 |
|  | A/Tunisia/9412/2022 | Tunisia | EPI2177198 | EPI2177197 | 2a.3b |
|  | A/Shanghai/FX1804C2/2022 | CHINA | EPI3589876 | EPI3589874 | 1a.1 |
|  | A/Myanmar/I039/2021 | Myanmar | EPI1998579 | EPI1998578 | 2a.3 |
|  | A/Brazil/BA/LACEN/BA064 292046737/2022 | Brazil | EPI2186320 | EPI2186319 | 2a.3 |
|  | A/Georgia/14289/2023 | USA | EPI2884355 | EPI2884354 | 2a.3a.1 |
|  | A/Canarias/230176/2022 | Spain | EPI2400126 | EPI2400125 | 2a.3a.1 |
|  | A/Puerto_Rico/45/2022 | Puerto Rico | EPI2347602 | EPI2347601 | 2a.1 |
|  | A/Sichuan/Beichuanqiangzuzizhi/36/2023 | China | EPI2669304 | EPI2669303 | 1a.1 |
|  | A/Denmark/747/2023 | Denmark | EPI2530234 | EPI2530233 | 2a.1b |
|  | A/Burkina_Faso/4677/2024 | Burkina Faso | EPI3607338 | EPI3607337 | 2a.3a |
|  | A/Mozambique/7967/2023 | Mozambique | EPI2677083 | EPI2677081 | 2B |
|  | A/Nepal/21FL3136/2021 | Nepal | EPI1998591 | EPI1998590 | 2a.3 |
|  | A/Gwangju/689/2023 | South Korea | EPI2454901 | EPI2454900 | 2a.3a.1 |
|  | A/Texas/111/2022 | USA | EPI3346872 | EPI3346871 | 2a.1a |
|  | A/Austria/MUW1581824/2023 | Austria | EPI2436547 | EPI2436546 | 2a.3a.1 |
|  | A/Vasteras/SE23/14213/2023 | Sweden | EPI3251461 | EPI3251460 | 2a.3a.1 |
|  | A/SouthSudan/631/2023 | South Sudan | EPI2760859 | EPI2762757 | 2a.3 |
|  | A/heilongjiangxi/An/1200/2023 | China | EPI2668810 | EPI2668809 | 2a.3a.1 |
|  | A/Thailand/8/2022 | Thailand | EPI2236266 | EPI2236265 | 2a.3a.1 |
|  | A/Queensland/IN000671/2024 | Australia | EPI3640509 | EPI3640507 | 2a.3a.1 |
|  | A/Nakhon_Ratchasima/THIS74/2023 | Thailand | EPI2793764 | EPI2793763 | 2a.3a.1 |
|  | A/Auckland/17/2024 | New Zealand | EPI3510416 | EPI3510410 | 2a.3a.1 |
|  | A/AbuDhabi/622842/2021 | U AE | EPI1881257 | EPI1885008 | 2d |
|  | A/Massachusetts/18/2022 | USA | EPI2413620 | EPI2413618 | 2a.3a.1 |
|  | A/Ecuador/1062/2021 | Ecuador | EPI2003445 | EPI2003444 | 2a |
|  | A/Cambodia/h1031363/2023 | Cambodia | EPI2976861 | EPI2976858 | 2a.3a.1 |
|  | A/Palencia/201/2022 | Spain | EPI2932394 | EPI2932393 | 2 b |
|  | A/Distrito_Federal/19614320/2022 | Brazil | EPI3042949 | EPI3042948 | 2a.3 |
|  | A/Birobidzhan/18/2024 | Russia | EPI3232013 | EPI3232012 | 2a.3a.1 |
|  | A/Malaysia/RP0963/2023 | Malaysia | EPI2976995 | EPI2976994 | 2a.3a.1 |
|  | A/Mecklenburg/Vorpommern/11/2022 | Germany | EPI2010833 | EPI2010832 | 2a.1 |
|  | A/Orebro/3/2022 | Sweden | EPI2220434 | EPI2220433 | 2a.3a.1 |
|  | A/Hunan/Tianyuan/1901/2023 | China | EPI2822284 | EPI2822283 | 1a.1 |
|  | A/Lisboa/80/2022 | Portugal | EPI2021273 | EPI2021272 | 2a.3a |
|  | A/Vietnam/Vnhcm/Vp235/2020 | Vietnam | EPI1847904 | EPI1847903 | 1a |
|  | A/Maryland/12400/2021 | USA | EPI1925992 | EPI1925991 | 2a.1a |
|  | A/Sundsvall/1/2022 | Sweden | EPI2020806 | EPI2020805 | 2a.1 |
|  | A/Heilongjiang/Saertu/1146/2024 | China | EPI3466808 | EPI3466807 | 2a.1 |
|  | A/Sao_Paulo/IAL/C9016/2022 | Brazil | EPI1997075 | EPI1997074 | 2a.3 |
|  | A/Croatia/10136RV/2023 | Croatia | EPI3472940 | EPI3472932 | 2a.3a.1 |
|  | A/Hungary/169/2024 | Hungary | EPI3447507 | EPI3447509 | 2a.3a.1 |
|  | A/Saudi Arabia/VRG/01/2016H3N2 | Saudi Arabia | ON514565 | ON514227 |  |
|  | A/Saudi Arabia/VRG/02/2016H3N2 | Saudi Arabia | ON514617 | ON514616 |  |
|  | A/Saudi Arabia/VRG/03/2016H3N2 | Saudi Arabia | ON520897 | ON520901 |  |
|  | A/Saudi Arabia/VRG/04/2017H3N2 | Saudi Arabia | ON521141 | ON521167 |  |
|  | A/Saudi Arabia/VRG/15/2017H3N2 | Saudi Arabia | ON521169 | ON521191 |  |
|  | A/Saudi Arabia/VRG/23/2017H3N2 | Saudi Arabia | ON521222 | ON521223 |  |
|  | A/Saudi Arabia/VRG/27/2017H3N2 | Saudi Arabia | ON522003 | ON524169 |  |
|  | A/Saudi Arabia/VRG/32/2017H3N2 | Saudi Arabia | ON524170 | ON524408 |  |
|  | A/Saudi Arabia/VRG/44/2017H3N2 | Saudi Arabia | ON627717 | ON524821 |  |
|  | A/Saudi Arabia/VRG/45/2017H3N2 | Saudi Arabia | ON524823 | ON524824 |  |
|  | A/Saudi Arabia/VRG/46/2018H3N2 | Saudi Arabia | ON524839 | ON524860 |  |
|  | A/Saudi Arabia/VRG/51/2018H3N2 | Saudi Arabia | ON524871 | ON525112 |  |
|  | A/Saudi Arabia/VRG/54/2018H3N2 | Saudi Arabia | ON527514 | ON527515 |  |
|  | A/Saudi Arabia/VRG/55/2018H3N2 | Saudi Arabia | ON527517 | ON527519 |  |
|  | A/Saudi Arabia/VRG/57/2018H3N2 | Saudi Arabia | ON527525 | ON527526 |  |
|  | A/Saudi Arabia/VRG/58/2018H3N2 | Saudi Arabia | ON527530 | ON527548 |  |
|  | A/Saudi Arabia/VRG/42/2020H3N2 | Saudi Arabia | OP536171 | OP536586 |  |
|  | A/Saudi Arabia/VRG/46/2020H3N2 | Saudi Arabia | OP536200 | OP536588 |  |
|  | A/Saudi Arabia/VRG/48/2020H3N2 | Saudi Arabia | OP536201 | OP536589 |  |
|  | A/Saudi Arabia/VRG/49/2020H3N2 | Saudi Arabia | OP536205 | OP536988 |  |
|  | A/Saudi Arabia/VRG/57/2020H3N2 | Saudi Arabia | OP536209 | OP537019 |  |
|  | A/Saudi Arabia/VRG/138/2020H3N2 | Saudi Arabia | OP536417 | OP537028 |  |
